# Enzalutamide + androgen deprivation therapy (ADT) versus flutamide + ADT in Japanese men with castration‐resistant prostate cancer: AFTERCAB study

**DOI:** 10.1002/bco2.103

**Published:** 2021-08-20

**Authors:** Hiroji Uemura, Kazuki Kobayashi, Akira Yokomizo, Shiro Hinotsu, Shigeo Horie, Yoshiyuki Kakehi, Seiji Naito, Norio Nonomura, Osamu Ogawa, Mototsugu Oya, Kazuhiro Suzuki, Atsushi Saito, Satoshi Uno, Hideyuki Akaza

**Affiliations:** ^1^ Yokohama City University Medical Center Yokohama Japan; ^2^ Yokosuka Kyosai Hospital Yokosuka Japan; ^3^ Harasanshin Hospital Fukuoka Japan; ^4^ Sapporo Medical University Sapporo Japan; ^5^ Graduate School of Medicine Juntendo University Tokyo Japan; ^6^ Department of Urology National University Corporation Kagawa University Takamatsu Japan; ^7^ Graduate School of Medicine Osaka University Osaka Japan; ^8^ Graduate School of Medicine Kyoto University Kyoto Japan; ^9^ School of Medicine Keio University Tokyo Japan; ^10^ Graduate School of Medicine Gunma University Maebashi Japan; ^11^ Astellas Pharma Inc. Tokyo Japan; ^12^ Department of Strategic Investigation on Comprehensive Cancer Network, Research Center for Advanced Science and Technology The University of Tokyo Tokyo Japan

**Keywords:** AFTERCAB, alternative androgen therapy, androgen deprivation therapy, bicalutamide, castration‐resistant prostate cancer, combined androgen blockade, enzalutamide, flutamide, Japan, prostate‐specific antigen progression

## Abstract

**Objectives:**

The objective of the study is to compare the efficacy and safety of alternative androgen therapy (AAT) with enzalutamide + androgen deprivation therapy (ADT) and flutamide + ADT in the treatment of Japanese men with metastatic or nonmetastatic castration‐resistant prostate cancer (CRPC) who progressed despite combined androgen blockade (CAB) with bicalutamide + ADT. AAT treatment sequence was also investigated.

**Materials and methods:**

The open‐label, Phase 4 AFTERCAB study (NCT02918968) was conducted from November 2016 to March 2020 in Japanese men aged ≥20 years with asymptomatic or mildly symptomatic CRPC. Patients were initially randomized to enzalutamide (160 mg/day) + ADT (enzalutamide first) or flutamide (375mg/day [125mg three times daily]) + ADT (flutamide first) as first‐line therapy. Following prostate‐specific antigen (PSA) progression, other disease progression, or discontinuation of first‐line therapy due to an adverse event (AE), patients switched to the other treatment as second‐line therapy. The primary endpoint was time to PSA progression with first‐line therapy (TTPP1). Secondary endpoints included TTPP2 (TTPP1 + time to PSA progression with second‐line therapy). AEs were monitored to assess safety.

**Results:**

Overall, 206 men were randomized (enzalutamide first, *n* = 102; flutamide first, *n* = 104) and stratified by study site and disease stage; 133 patients transitioned to second‐line therapy (enzalutamide first, *n* = 48; flutamide first, *n* = 85). TTPP1 was significantly improved with enzalutamide first versus flutamide first (median 21.4 months vs. 5.8 months; hazard ratio [HR] 0.42; 95% confidence interval [CI] [0.29, 0.61]). TTPP2 was numerically improved with enzalutamide first versus flutamide first (median not reached vs. 21.2 months; HR 0.76; 95% CI [0.48, 1.19]). Both treatments were generally well tolerated, with AEs consistent with their known safety profiles.

**Conclusion:**

First‐line AAT with enzalutamide + ADT provided a significant improvement in time to PSA progression versus flutamide + ADT. Enzalutamide + ADT may therefore be the preferred first‐line AAT option in Japanese men with metastatic or nonmetastatic CRPC who progress despite CAB with bicalutamide + ADT.

## 1 INTRODUCTION

In 2018, the global incidence of prostate cancer was estimated at 1 276 106, based on the World Health Organization's GLOBOCAN project.^1^ The highest incidence of prostate cancer was reported in Europe, with 449 761 cases, followed by Asia, with 297 215 cases.^1^ The incidence in Japan was estimated to be 78 400, with prostate cancer accounting for an estimated 12 400 deaths in Japanese men and ranking sixth with respect to cancer‐related mortality.^1^, ^2^

Bicalutamide + androgen deprivation therapy (ADT), herein referred to as combined androgen blockade (CAB), is widely used for the treatment of metastatic and nonmetastatic prostate cancer in Japan, owing to the observed long‐term efficacy.^3^, ^4^, ^5^, ^6^ However, a proportion of patients receiving CAB experience prostate‐specific antigen (PSA) recurrence and progress to castration‐resistant prostate cancer (CRPC). Based on the Japanese Urological Association 2012 Prostate Cancer Guidelines, alternative androgen therapy (AAT) with flutamide + ADT is recommended for the treatment of CRPC in Japanese patients who progress despite CAB^7^ and is often used in clinical practice.^8^, ^9^, ^10^, ^11^, ^12^, ^13^, ^14^ However, additional AAT options are required given the modest PSA benefit observed with flutamide in some patients.

Enzalutamide is a potent oral androgen receptor inhibitor that is either approved or under regulatory consideration for approval in countries around the world for the treatment of men with metastatic hormone‐sensitive prostate cancer (also known as metastatic castration‐sensitive prostate cancer) and CRPC, irrespective of the presence of metastases.^15^, ^16^, ^17^ Enzalutamide, with concomitant surgical or medical castration, has been approved by Japan's Ministry of Health, Labour, and Welfare for the treatment of men with CRPC since 2014,^18^ based on multiple studies,^19^, ^20^, ^21^ and was approved in men with prostate cancer with distant metastases in 2020,^18^ based on the ARCHES^22^ and ENZAMET^23^ trials. Enzalutamide may, therefore, offer an additional first‐line AAT option in Japanese men with CRPC who experience PSA progression despite CAB.

The OCUU‐CRPC study was the first to compare enzalutamide versus flutamide as first‐line AAT in Japanese men with CRPC following CAB with bicalutamide + ADT.^24^, ^25^ Improved clinical outcomes were observed following treatment with enzalutamide, suggesting that enzalutamide may provide a preferable first‐line AAT option.^25^ However, cross‐resistance between androgen therapies is a known obstacle in the treatment of CRPC,^26^ and the preferable treatment sequence of AAT is yet to be determined. Here, we present the results of the AFTERCAB study, which aimed to compare the efficacy and safety of enzalutamide + ADT and flutamide + ADT in Japanese men with metastatic or nonmetastatic CRPC who progressed despite CAB with bicalutamide + ADT. The optimal sequential order of AAT was also investigated.

## 2 MATERIALS AND METHODS

### 2.1 Study design

AFTERCAB (NCT02918968) was a randomized, open‐label, Phase 4, post‐marketing clinical study, conducted between November 2016 and March 2020 in Japanese men with metastatic or nonmetastatic CRPC who progressed despite CAB with bicalutamide + ADT. The study was conducted in accordance with International Council for Harmonisation guidelines, applicable local laws, regulations, and guidelines governing clinical study conduct, and ethical principles that have their origin in the Declaration of Helsinki.

**FIGURE 1:**
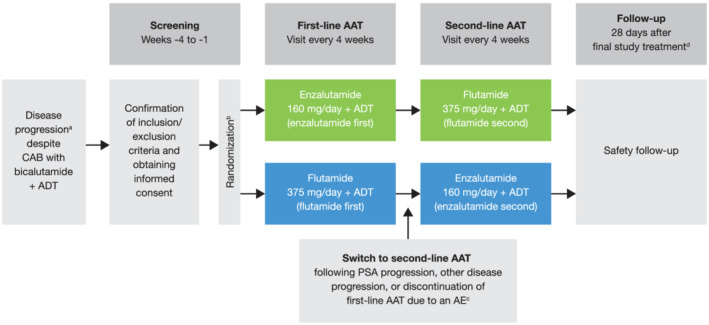
Study design. ^a^Disease progression, defined as a confirmed prostate‐specific antigen (PSA) increase above the highest PSA measured after nadir during bicalutamide treatment, measured ≥6 weeks after the last dose of bicalutamide, or soft tissue disease progression defined according to the Response Evaluation Criteria in Solid Tumors guidelines (version 1.1),^27^ or progression of ≥2 new bone lesions identified via bone scintigraphy according to the Prostate Cancer Working Group 2 guidelines^28^; ^b^Randomization stratified by study site and disease stage, as M0/N0: No distant metastases; no lymph node metastases; M0/N1: No distant metastases; with lymph node metastases distal to the aortic bifurcation; or M1: With distant metastases, including lymph node metastases proximal to the aortic bifurcation; ^c^Patients who discontinued first‐line alternative androgen therapy (AAT) due to PSA progression started second‐line AAT within 6 weeks of the date of examination; patients who discontinued first‐line AAT due to other disease progression or an AE started second‐line AAT within 6 weeks of the date of investigator decision to discontinue; ^d^28 days after final study treatment or before starting other study drugs or before starting other treatment for prostate cancer, whichever comes first. During both first‐ and second‐line AAT, PSA was measured at screening, Week 1, Week 13, Week 17, and every subsequent 4 weeks. PSA was also measured at final study treatment or discontinuation, and at follow‐up (28 days after final study treatment). Unscheduled PSA measurement was permitted if considered required by the study investigator. During both first‐ and second‐line AAT, abdominopelvic computed tomography (CT)/magnetic resonance imaging, bone scintigraphy, and chest CT imaging was performed at screening, Week 13, and every subsequent 12 weeks. Imaging was also performed at final study treatment or discontinuation, and unscheduled imaging was permitted if considered required by the study investigator. Chest CT was not required in patients with no chest metastases identified at screening

Patients were initially randomized 1:1 to enzalutamide + ADT (enzalutamide first) or flutamide + ADT (flutamide first) as first‐line therapy, stratified by study site and disease stage, as M0/N0, M0/N1, or M1 (Figure 1). Enzalutamide was administered as 160 mg/day and flutamide as 375 mg/day (125 mg three times daily), as instructed on the package inserts.

Following confirmation of PSA progression, other disease progression, or discontinuation of first‐line therapy due to an adverse event (AE), patients switched to the other treatment arm as second‐line therapy within 6 weeks of the date of examination or investigator decision. PSA progression was defined according to the Prostate Cancer Working Group 2 guidelines.^28^ For men with PSA decline at Week 13, PSA progression was defined as a ≥25% increase and an absolute increase of ≥2 ng/ml above the nadir, confirmed by a second consecutive value ≥3 weeks later. For men without PSA decline at Week 13, PSA progression was defined as a ≥25% increase and an absolute increase of ≥2 ng/ml above baseline. The treatment period with each therapy did not exceed 2 years from the enrollment of the last patient.

Treatment adjustment was permitted in the event of a National Cancer Institute Common Terminology Criteria for Adverse Events (CTCAE) Grade ≥3 AE, whereby treatment was stopped for 1 week or until the AE reduced in severity to Grade ≤2, after which treatment was re‐started at the original or reduced dose, at the investigator's discretion. No increases in study drug dose were permitted. Treatment was discontinued in the event of PSA progression, other disease progression, or intolerable AEs that were not improved by medical intervention or dose reduction. Treatment was also discontinued in the event of a seizure; however, men who had a seizure during first‐line treatment with enzalutamide were permitted to transition to second‐line treatment with flutamide. In addition, aspartate aminotransferase (AST) or alanine aminotransferase (ALT) greater than three times the upper limit of normal resulted in treatment discontinuation; however, men who experienced such a liver disorder during first‐line treatment with flutamide were permitted to transition to second‐line treatment with enzalutamide.

### 2.2 Patients

Men aged ≥20 years with asymptomatic or mildly symptomatic metastatic or nonmetastatic CRPC with disease progression despite CAB with bicalutamide + ADT were eligible for inclusion. All men were required to have histologically or cytologically confirmed adenocarcinoma of the prostate and to be receiving continuous ADT throughout the duration of the study. Men were also required to have an Eastern Cooperative Oncology Group performance status of 0 or 1, estimated life expectancy of ≥12 months, and a serum testosterone level of ≤1.73 nmol/L at screening.

Men with confirmed or suspected brain metastasis or active leptomeningeal metastasis were excluded, as were those with unstable psychiatric disease, history of seizure, or liver disorders such as viral hepatitis or hepatic cirrhosis. Men were not eligible if they had received any of the following prior treatments: bicalutamide within 6 weeks of enrollment; cytotoxic chemotherapy, abiraterone, or estramustine; surgery or radiation therapy for primary or metastatic lesion within 4 weeks prior to enrollment; opioid analgesics for pains associated with prostate cancer within 4 weeks prior to enrollment; or 5‐α reductase inhibitors, estrogens, or drugs with anti‐tumor action (other than gonadotropin‐releasing hormone agonists/antagonists) within 4 weeks prior to enrollment.

All eligible men were required to provide informed consent, approved by the institutional review board at each study center, prior to the study. Full details of all inclusion and exclusion criteria are provided in Table S1.

### 2.3 Endpoints

The primary endpoint was time to PSA progression with first‐line therapy (TTPP1). PSA progression was defined as previously described. Secondary endpoints were time to PSA progression with first‐line therapy + second‐line therapy (TTPP2), PSA response rate (≥50% or ≥90%) with first‐line therapy, PSA response rate (≥50% or ≥90%) with first‐line therapy at Week 13, time to 50% PSA reduction with first‐line therapy, time to treatment failure with first‐line therapy (TTF1), time to treatment failure with second‐line therapy (TTF2), and radiographic progression‐free survival (rPFS; in men with distant metastases confirmed at baseline). Exploratory endpoints included time to PSA progression with second‐line therapy (second TTPP) and PSA response rate (≥50% or ≥90%) with second‐line therapy. The full definitions of all efficacy endpoints are provided in Table S2.

AEs were monitored until 28 days after the final treatment with study drug to assess safety.

### 2.4 Statistical analyses

Sample size was calculated using an expected median TTPP1 of 10.5 months with enzalutamide, based on a subgroup analysis of Japanese patients (data on file) from the PREVAIL study, and 6 months with flutamide.^10^, ^30^ Using a log‐rank‐based method^31^ with a planned enrollment period of 12 months and planned observation period of 24 months from the enrollment of the last patient, 135 TTPP1 events were required to provide a two‐sided error rate of 0.05 and power of 90%. By allowing for a drop‐out rate of ~25%, 100 patients were planned to be enrolled in each treatment arm.

Efficacy analyses were performed on the intent‐to‐treat population, defined as all randomized patients. Safety analyses were performed on the safety analysis set (SAF), defined as all patients who received at least one dose of study drug.

Data were summarized using descriptive statistics for continuous endpoints, and frequency and percentage for categorical endpoints. Hypothesis testing for group comparison for TTPP1, time to 50% PSA reduction, and TTF1 was assessed via stratified log‐rank test, with disease stage as the stratification factor. A secondary analysis of TTPP1 and post hoc analysis of TTPP2 were also performed using an unstratified Cox's proportional hazards model, with disease stage as a covariate. The distribution of TTPP1, TTPP2, time to 50% PSA reduction, TTF1, TTF2, and second TTPP were estimated using the Kaplan–Meier method. PSA response rate with its Clopper–Pearson exact confidence interval was calculated by treatment group. Comparison of the response rate was calculated as Mantel–Haenszel common risk difference using the stratified Cochran–Mantel–Haenszel test,^32^ with disease stage as the stratification factor. rPFS was assessed using the same method as the primary analysis of TTPP1 in men with distant metastases confirmed at baseline. For all analyses, within the exception of TTPP2, second TTPP, and TTF2, for which no hypothesis testing was planned, the significance level was 0.05 (two‐sided).

AEs were coded using the Medical Dictionary for Regulatory Activities, version 23.0. Severity of AEs was evaluated by the investigator, based on CTCAE, version 4.0.

## 3 RESULTS

**FIGURE 2:**
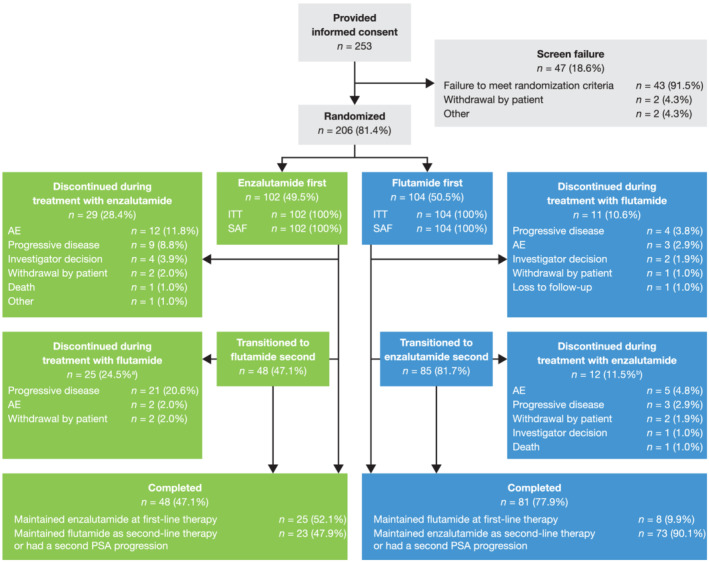
Patient disposition. ^a^Relative to number of patients randomized to enzalutamide first; ^b^Relative to number of patients randomized to flutamide first

Patient disposition is presented in Figure 2. In total, 206 men were randomized and comprised the intent‐to‐treat population and SAF. Of the 102 men randomized to enzalutamide first, 29 discontinued during first‐line therapy with enzalutamide and 48 transitioned to second‐line therapy with flutamide; 25 men maintained first‐line therapy with enzalutamide until the end of the study, while 23 men maintained flutamide as second‐line therapy or had a second PSA progression. Of the 104 men randomized to flutamide first, 11 discontinued during first‐line therapy with flutamide and 85 transitioned to second‐line therapy with enzalutamide; 8 men maintained first‐line therapy with flutamide until the end of the study, while 73 men maintained enzalutamide as second‐line therapy or had a second PSA progression. AEs were the most common reasons for discontinuation during treatment with enzalutamide, while progressive disease was the most common reason for discontinuation during treatment with flutamide.

Baseline demographics and disease characteristics were similar between treatment arms with the exception of Brief Pain Inventory, Short Form, Question 3 score, for which a slightly greater proportion of men receiving enzalutamide first had a score of 0–1 compared with men receiving flutamide first (90.2% vs. 78.8%) (Table 1).

**TABLE 1:** Baseline demographics and disease characteristics

Parameter	Enzalutamide first (*n* = 102)	Flutamide first (*n* = 104)
Mean age ± SD, years	74.4 ± 7.6	74.1 ± 7.6
Mean weight ± SD, kg	67.7 ± 10.1	66.9 ± 9.9
Mean BMI ± SD, km/m^2^	25.2 ± 3.3	24.8 ± 2.8
Median (range) serum PSA, ng/ml	8.5 (2.1–663.6)	7.6 (2.1–731.1)
ECOG performance status 0, *n* (%)	91 (89.2)	90 (86.5)
BPI‐SF question 3 score 0–1, *n* (%)	92 (90.2)	82 (78.8)
Disease stage at randomization, *n* (%)		
M0/N0	24 (23.5)	25 (24.0)
M0/N1	3 (2.9)	4 (3.8)
M1	75 (73.5)	75 (72.1)
Distant metastasis at randomization, *n* (%)		
M0	27 (26.5)	29 (27.9)
M1	75 (73.5)	75 (72.1)
Regional lymph nodes at randomization, *n* (%)		
N0	75 (73.5)	81 (77.9)
N1	27 (26.5)	23 (22.1)
Median (range) time from diagnosis, months	26.0 (6.2–222.4)	25.9 (5.3–188.9)
Gleason score category at diagnosis, *n* (%)		
Low (2–4)	0	0
Medium (5–7)	14 (13.7)	19 (18.3)
High (8–10)	84 (82.4)	84 (80.8)
Missing	4 (3.9)	1 (1.0)
Median (range) PSA doubling time, months	2.4 (0.2–72.8)	2.3 (0.5–49.3)
Median (range) bicalutamide treatment duration, months	18.5 (3.1–169.2)	16.8 (3.0–185.4)

Abbreviations: BMI, body mass index; BPI‐SF, Brief Pain Inventory, Short Form; ECOG, Eastern Cooperative Oncology Group; M0, no distant metastases; M1, with distant metastases, including lymph node metastases proximal to the aortic bifurcation; N0, no lymph node metastases; N1, with lymph node metastases distal to the aortic bifurcation; SD, standard deviation.

Median (range) duration of exposure was 14.3 months (0.8–35.9) with enzalutamide as first‐line therapy and 2.7 months (0.2–9.2) with flutamide as second‐line therapy. Meanwhile, median (range) duration of exposure was 5.6 months (0.3–37.7) with flutamide as first‐line therapy and 9.2 months (0.9–33.9) with enzalutamide as second‐line therapy.

### 3.1 Primary endpoint

**FIGURE 3:**
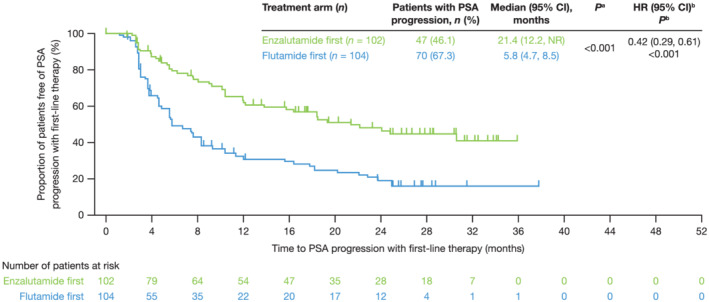
TTPP1. Time from randomization to the date of prostate‐specific antigen (PSA) progression with first‐line therapy. PSA progression was defined according to the Prostate Cancer Working Group 2 guidelines.^28^ For patients with PSA decline at Week 13, PSA progression was defined as a ≥25% increase and an absolute increase of ≥2 ng/ml above the nadir, confirmed by a second consecutive value ≥3 weeks later. For patients without PSA decline at Week 13, PSA progression was defined as a ≥25% increase and an absolute increase of ≥2 ng/ml above baseline. ^a^Stratified log‐rank test, stratified by disease stage; ^b^Unstratified Cox proportional hazards model, with treatment and disease stage as covariates

TTPP1 was significantly longer in men receiving enzalutamide first versus those receiving flutamide first, based on a stratified log‐rank test. Median (95% confidence interval [CI]) TTPP1 was 21.4 months [12.2, not reached (NR)] and 5.8 months [4.7, 8.5], respectively. Results of the secondary analysis using an unstratified Cox's proportional hazards model were consistent (hazard ratio [HR] 0.42; 95% CI [0.29, 0.61]) [Figure 3].

### 3.2 Secondary endpoints

**FIGURE 4:**
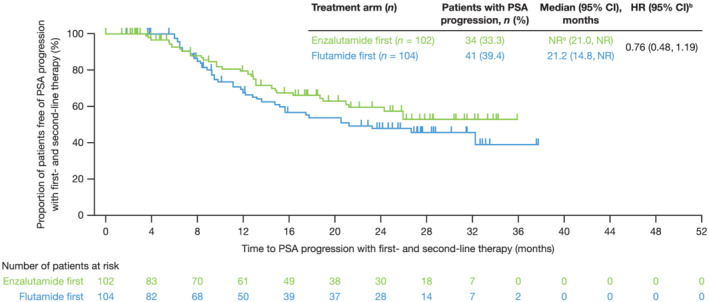
TTPP2. Total of time to prostate‐specific antigen (PSA) progression with first‐line therapy (time from randomization to the date of PSA progression with first‐line therapy) and time to PSA progression with second‐line therapy (time from Day 1 of second‐line therapy to the date of PSA progression with second‐line therapy). PSA progression was defined according to the Prostate Cancer Working Group 2 guidelines.^28^ For patients with PSA decline at Week 13, PSA progression was defined as a ≥25% increase and an absolute increase of ≥2 ng/ml above the nadir, confirmed by a second consecutive value ≥3 weeks later. For patients without PSA decline at Week 13, PSA progression was defined as a ≥25% increase and an absolute increase of ≥2 ng/ml above baseline. ^a^At a maximum observation of 35.9 months; ^b^Post hoc analysis using unstratified Cox proportional hazards model, with treatment and disease stage as covariates

Median [95% CI] TTPP2 was numerically longer with enzalutamide first, compared with flutamide first: NR at a maximum observation of 35.9 months (21.0, NR) compared with 21.2 months (14.8, NR); HR 0.76; 95% CI [0.48, 1.19] (Figure 4).

**FIGURE 5:**
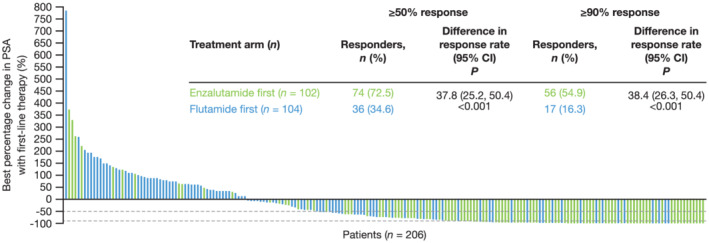
Prostate‐specific antigen (PSA) response rate (≥50% or ≥90%) with first‐line therapy. Proportion of patients who achieved PSA response with first‐line therapy. PSA response defined as a ≥50% or ≥90% reduction from baseline when ≥3 weeks passed after the lowest PSA, which decreased by ≥50% or ≥90%

PSA response rates with first‐line therapy were significantly higher with enzalutamide first versus flutamide first. Overall, 72.5% of men receiving enzalutamide first and 34.6% of men receiving flutamide first had ≥50% PSA response, and the difference in response rate [95% CI] was 37.8% [25.2, 50.4]. Corresponding values for ≥90% PSA response were 54.9% and 16.3%, respectively, with a 38.4% [26.3, 50.4] difference in response rate (Figure 5). Findings for PSA response rate with first‐line therapy at Week 13 were similar, with 74.5% of men receiving enzalutamide first and 33.7% of men receiving flutamide first having ≥50% PSA response, and a 40.7% [28.3, 53.1] difference in response rate. Corresponding values for ≥90% PSA response were 49.0% and 15.4%, respectively, with a 33.5% [21.5, 45.4] difference in response rate (Figure S1).

**FIGURE 6:**
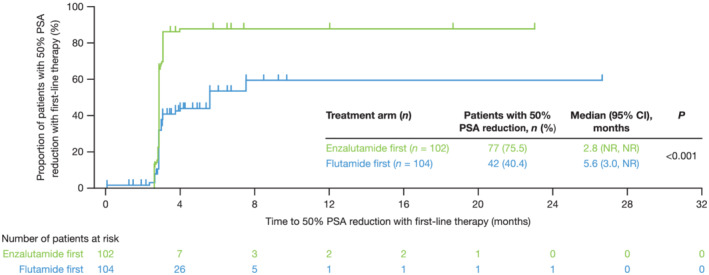
Time to 50% prostate‐specific antigen (PSA) reduction with first‐line therapy. Time to 50% PSA reduction from baseline with first‐line therapy [Correction added on 16 Oct 2021 after first online publication: In figure 6, progression was changed to reduction in this version.]

Time to 50% PSA reduction with first‐line therapy [95% CI] was significantly shorter for enzalutamide first, compared with flutamide first: 2.8 months [NR, NR] compared with 5.6 months [3.0, NR] (Figure 6).

TTF1 [95% CI] was significantly longer with enzalutamide first, compared with flutamide first: 13.8 months [9.2, 18.4] compared with 4.6 months [3.7, 5.8] (Figure S2). TTF2 (95% CI) was numerically longer in men randomized to enzalutamide first, compared with flutamide first: 23.0 months [16.8, NR] compared with 16.6 months [13.1, 24.0] (Figure S3).

No significant difference in rPFS was observed between men with distant metastases receiving enzalutamide first and those receiving flutamide first (median NR for either treatment arm); however, these results should be interpreted with caution due to low numbers of patients and events (Figure S4).

### 3.3 Exploratory endpoints

Second TTPP [95% CI] was numerically longer in men randomized to flutamide first who received enzalutamide as second‐line therapy, compared with those randomized to enzalutamide first who received flutamide as second‐line therapy: 10.2 months [7.2, 12.0] compared with 2.6 months [2.6, 2.8] (Figure S5).

PSA response rates (≥50% or ≥90%) with second‐line therapy were significantly higher in men randomized to flutamide first who received enzalutamide as second‐line therapy, compared with those randomized to enzalutamide first who received flutamide as second‐line therapy; 55.3% and 2.1%, respectively, for ≥50% response rate with a corresponding difference [95% CI] of 53.8% [43.1, 64.5]. Values for ≥90% PSA response were 29.4% and 2.1%, respectively, with a corresponding difference [95% CI] of 27.4% [17.0, 37.9] (Figure S6).

### 3.4 Safety

During first‐line therapy, 33.3% of men receiving enzalutamide first and 11.5% of men receiving flutamide first had at least one dose reduction or interruption. During second‐line therapy with flutamide, 4.9% of men had at least one dose reduction or interruption. Also, 21.2% of men reported at least one dose reduction or interruption while receiving second‐line therapy with enzalutamide. Dose reductions or interruptions were predominantly caused by AEs.

During first‐line therapy, 92.2% of men receiving enzalutamide first and 76.0% of men receiving flutamide first experienced an AE. The proportion of men experiencing AEs leading to discontinuation, drug‐related AEs, CTCAE Grade ≥3 AEs, serious AEs (SAEs), SAEs leading to discontinuation, and drug‐related SAEs was higher with enzalutamide first, compared with flutamide first. The safety profiles of second‐line therapies were generally similar to the safety profiles of those treatments when administered as first‐line therapy (Table 2). One man in the enzalutamide first treatment arm died due to prostate cancer during first‐line therapy with enzalutamide, and one man in the flutamide first treatment arm died due to a cerebellar hemorrhage during second‐line therapy with enzalutamide; neither event was considered to be related to the study drug by the investigator.

**TABLE 2:** Overview of treatment‐emergent adverse events

*n* (%)	First‐line therapy		Second‐line therapy	
	Enzalutamide (enzalutamide first) *n* = 102	Flutamide (flutamide first) *n* = 104	Flutamide (randomized to enzalutamide first) *n* = 48	Enzalutamide (randomized to flutamide first) *n* = 85
AEs	94 (92.2)	79 (76.0)	34 (70.8)	74 (87.1)
AEs leading to discontinuation	19 (18.6)	14 (13.5)	3 (6.3)	7 (8.2)
Drug‐related AEs	67 (65.7)	46 (44.2)	12 (25.0)	50 (58.8)
CTCAE Grade ≥3 AEs	45 (44.1)	17 (16.3)	6 (12.5)	22 (25.9)
SAEs	29 (28.4)	15 (14.4)	4 (8.3)	18 (21.2)
SAEs leading to discontinuation	14 (13.7)	3 (2.9)	1 (2.1)	3 (3.5)
Drug‐related SAEs	7 (6.9)	4 (3.8)	0	5 (5.9)
SAEs leading to death	1 (1.0)^a^	0	0	1 (1.2)^b^
AEs reported in ≥5% of patients in any treatment line				
Nasopharyngitis	23 (22.5)	17 (16.3)	2 (4.2)	12 (14.1)
Fall	18 (17.6)	5 (4.8)	1 (2.1)	10 (11.8)
Back pain	15 (14.7)	6 (5.8)	1 (2.1)	4 (4.7)
Malaise	15 (14.7)	4 (3.8)	2 (4.2)	17 (20.0)
Fatigue	15 (14.7)	3 (2.9)	0	8 (9.4)
Constipation	14 (13.7)	3 (2.9)	0	2 (2.4)
Hypertension	13 (12.7)	2 (1.9)	0	5 (5.9)
Decreased appetite	11 (10.8)	4 (3.8)	2 (4.2)	8 (9.4)
Diarrhea	7 (6.9)	12 (11.5)	6 (12.5)	2 (2.4)
Nausea	7 (6.9)	5 (4.8)	2 (4.2)	6 (7.1)
Influenza	7 (6.9)	2 (1.9)	0	1 (1.2)
Spinal compression fracture	7 (6.9)	0	1 (2.1)	2 (2.4)
Cancer pain	6 (5.9)	4 (3.8)	4 (8.3)	6 (7.1)
Dizziness	6 (5.9)	3 (2.9)	0	5 (5.9)
Hematuria	6 (5.9)	2 (1.9)	0	2 (2.4)
Hot flush	4 (3.9)	6 (5.8)	0	1 (1.2)
Anemia	4 (3.9)	2 (1.9)	4 (8.3)	1 (1.2)
Abnormal hepatic function	3 (2.9)	10 (9.6)	2 (4.2)	4 (4.7)
Increased ALT	2 (2.0)	7 (6.7)	1 (2.1)	0
Increased AST	2 (2.0)	6 (5.8)	2 (4.2)	0
Dental caries	1 (1.0)	7 (6.7)	1 (2.1)	5 (5.9)

Abbreviations: AE, adverse event; ALT, alanine aminotransferase; AST, aspartate aminotransferase; CTCAE, National Cancer Institute Common Terminology Criteria for Adverse Events; SAE, serious adverse event.

^a^ Due to prostate cancer.

^b^ Due to cerebellar hemorrhage.

The most commonly reported AE with first‐line therapy was nasopharyngitis, reported by 22.5% of men receiving enzalutamide first and 16.3% of men receiving flutamide first. In general, the incidence of AEs was higher in men receiving enzalutamide first, with the exception of diarrhea, hot flush, abnormal hepatic function, increased ALT, increased AST, and dental caries, which were higher in men receiving flutamide first. The incidence of malaise increased from 3.8% in men randomized to flutamide first to 20.0% after treatment was switched to enzalutamide. Similarly, the incidence of fatigue increased from 2.9% to 9.4%, and the incidence of hypertension increased from 1.9% to 5.9% following the transition from flutamide to enzalutamide (Table 2). No seizure or interstitial pneumonia AEs were reported. During second‐line therapy with enzalutamide, three men (3.5%) experienced decreased platelet count, of which two were CTCAE Grade ≥3. While ALT and AST elevations were generally more common in men receiving flutamide, one man had ALT and AST > 10 times the upper limit of normal during second‐line therapy with enzalutamide.

## 4 DISCUSSION

In this Phase 4, post‐marketing clinical study in Japanese men with metastatic or nonmetastatic CRPC who progressed despite CAB with bicalutamide + ADT, treatment with enzalutamide + ADT as first‐line AAT provided a significant improvement in TTPP1 versus flutamide + ADT. Across multiple PSA outcomes, a more favorable efficacy profile was observed with enzalutamide, whether received as first‐ or second‐line therapy. rPFS was similar between treatment arms in men with distant metastases at baseline; however, low numbers of events limit interpretability of results. Both treatments were generally well tolerated, with AEs consistent with the known safety profiles and no seizure or interstitial pneumonia AEs reported. Despite the incidence of AEs being higher during treatment with enzalutamide, patients spent longer on enzalutamide as both first‐ and second‐line therapies, as demonstrated by longer TTF1, TTF2, and treatment exposure, suggesting that an overall benefit of enzalutamide treatment can be expected.

In the previously published OCUU‐CRPC study, both 3‐ and 6‐month ≥50% PSA response rates were observed to be significantly higher in patients receiving enzalutamide, compared with those receiving flutamide (3‐month: 80.8% vs. 35.3%; 6‐month: 73.1% vs. 31.4%).^25^ These findings are consistent with the observed significant improvement in ≥50% PSA response rate with first‐line therapy (72.5% for enzalutamide first vs. 34.6% for flutamide first) and ≥50% PSA response rate at Week 13 with first‐line therapy (74.5% vs. 33.7%) in the AFTERCAB study.

Cross‐resistance between androgen therapies is a known obstacle in the treatment of CRPC.^26^ Indeed, in our study, the proportion of men achieving ≥50% PSA response rate decreased from 72.5% with first‐line therapy to 55.3% with second‐line for enzalutamide, and from 34.6% to 2.1% for flutamide. A similar effect was observed for ≥90% PSA response rates. In addition, time to PSA progression was shorter with second‐line therapy for both treatment arms (TTPP1 vs. second TTPP: 21.4 months vs. 10.2 months with enzalutamide; 5.8 months vs. 2.6 months with flutamide). Similarly, an increase of 15.4 months free of PSA progression from median TTPP1 (5.8 months) to median TTPP2 (21.2 months) with flutamide first can be attributed to enzalutamide, but it did not exceed median TTPP1 with enzalutamide first (21.4 months). In addition, TTF2 was numerically longer with enzalutamide first (23.0 months vs. 16.6 months with flutamide first). Therefore, it may be considered that early initiation of enzalutamide as first‐line AAT is preferable. While median TTPP2 was NR with enzalutamide first, a numerically longer duration of TTPP2 was observed compared with flutamide first, which was consistent with the HR of 0.76 observed in the post hoc analysis.

The provision of long‐term follow‐up data related to both first‐ and second‐line AAT from a randomized, well‐controlled setting represents a key strength of the AFTERCAB study. In addition, the analysis of PSA‐related endpoints is beneficial, given the regularity of PSA assessment during routine clinical practice in Japan. However, the following inherent limitations should be considered. First, this was an open‐label study that did not investigate overall or cancer‐specific survival. In addition, the number of patients who transitioned to second‐line therapy was imbalanced between treatment arms as a result of both efficacy and safety considerations. A slightly higher number of patients who received enzalutamide first discontinued due to AEs; otherwise, there were more patients randomized to enzalutamide first who continued first‐line therapy until the end of the study without PSA progression. This resulted in difficulties with the interpretation of efficacy endpoints related to second‐line therapy, such as TTPP2, as the balance of patient characteristics ensured by baseline randomization was potentially not sustained. In addition, incidence of AEs was calculated as the proportion of men from the SAF who had experienced at least one treatment‐emergent AE, and therefore, adjustment for observation length was not considered. The increased treatment duration with enzalutamide may therefore have contributed to the higher incidence of AEs observed in patients receiving enzalutamide. Additional analyses of this study population, related to healthcare resource utilization, would be beneficial to further inform the use of enzalutamide + ADT as first‐line AAT in the Japanese healthcare system.

In conclusion, treatment with enzalutamide + ADT as first‐line AAT provided a significant improvement in time to PSA progression and other PSA‐related outcomes versus flutamide + ADT as first‐line therapy. Enzalutamide + ADT as first‐line AAT may therefore be the preferred treatment option for Japanese men with metastatic or nonmetastatic CRPC who progress despite CAB with bicalutamide + ADT. Additional large studies of comparable design are needed to confirm our findings and add to the current body of evidence regarding the optimal sequential order of AAT.

## CONFLICT OF INTEREST

Hiroji Uemura reports personal fees from Astellas during the conduct of the study and consulting fees from Amgen, Astellas, AstraZeneca, and Janssen; consulting fees and research funding from Daiichi Sankyo; and research funding from Bayer, Chugai, Kissei, Sanofi, Taiho, and Takeda outside of the submitted work. Kazuki Kobayashi reports grants from Astellas during the conduct of the study. Akira Yokomizo reports grants from Astellas during the conduct of the study and lecture fees from Astellas, Bayer, and Sanofi outside of the submitted work. Shiro Hinotsu reports his role as a study investigator during the conduct of the study. Shigeo Horie reports grants from Astellas, Nippon Shinyaku, and Sanofi and personal fees from Astellas, AstraZeneca, Nippon Shinyaku, and Sanofi during the conduct of the study. Yoshiyuki Kakehi reports consulting fees from Astellas during the conduct of the study and outside of the submitted work. Seiji Naito reports his role as a study investigator during the conduct of the study and consulting fees from Astellas outside of the submitted work. Norio Nonomura reports his role as a study investigator during the conduct of the study and consulting fees from Astellas outside of the submitted work. Osamu Ogawa reports nonfinancial support from Astellas during the conduct of the study and personal fees from Astellas outside of the submitted work. Mototsugu Oya reports grants from Astellas and personal fees from Astellas, AstraZeneca, Bayer, Janssen, Sanofi, and Takeda during the conduct of the study. Kazuhiro Suzuki reports nonfinancial support from Astellas during the conduct of the study, and consulting fees and honoraria from AstraZeneca and Bayer Yakuhi, grants, consulting fees, and honoraria from Astellas and Takeda, grants and honoraria from Chugai Pharma, Daiichi Sankyo, and Nippon Shinyaku, honoraria from Janssen, Merck, MSD, Ono Pharmaceutical, and Pfizer, and grants from Kyowa Hakko Kirin and Sanofi outside of the submitted work. Atsushi Saito and Satoshi Uno are employees of Astellas Pharma Inc. Hideyuki Akaza reports his role as a study investigator during the conduct of the study, and grants from Astellas and Takeda outside of the submitted work.

## Supporting information


**Data S1:** Supporting information.

## Data Availability

Researchers may request access to anonymized participant‐level data, trial‐level data, and protocols from Astellas‐sponsored clinical trials online (www.clinicalstudydatarequest.com). For the Astellas criteria on data sharing see online (https://clinicalstudydatarequest.com/Study-Sponsors/Study-Sponsors-Astellas.aspx).

## References

[1] World Health Organization . GLOBOCAN cancer today: prostate cancer fact sheet, 2019. Available at: http://gco.iarc.fr/today/data/factsheets/cancers/27-Prostate-fact-sheet.pdf. Accessed 2 November 2020

[2] National Cancer Center for Cancer Control and Information Services . Cancer Statistics in Japan '18, 2019. Available at: https://ganjoho.jp/en/professional/statistics/brochure/2018_en.html. Accessed November 2020

[3] Akaza H , Hinotsu S , Usami M , et al. Combined androgen blockade with bicalutamide for advanced prostate cancer: long‐term follow‐up of a Phase 3, double‐blind, randomized study for survival. Cancer. 2009;115:3437–3445. 19536889 10.1002/cncr.24395

[4] Onozawa M , Akaza H , Hinotsu S , et al. Combined androgen blockade achieved better oncological outcome in androgen deprivation therapy for prostate cancer: Analysis of community‐based multi‐institutional database across Japan using propensity score matching. Cancer Med. 2018;7:4893–4902. 30151999 10.1002/cam4.1735 PMC6198209

[5] Ueno S , Namiki M , Fukagai T , Ehara H , Usami M , Akaza H . Efficacy of primary hormonal therapy for patients with localized and locally advanced prostate cancer: a retrospective multicenter study. Int J Urol. 2006;13:1494–1500. 17118024 10.1111/j.1442-2042.2006.01604.x

[6] Kakehi Y , Sugimoto M , Taoka R , Committee for establishment of the evidenced‐based clinical practice guideline for prostate cancer of the Japanese Urological Association . Evidenced‐based clinical practice guideline for prostate cancer (summary: Japanese Urological Association, 2016 edition). Int J Urol. 2017;24:648–6666. 28667698 10.1111/iju.13380

[7] The Japanese Urological Association . Prostate cancer clinical practice guideline 2012 [In Japanese]. Tokyo: Kanehara‐&Co., Ltd; 2012.

[8] Yasui M , Uemura K , Yoneyama S , et al. Predictors of poor response to secondary alternative antiandrogen therapy with flutamide in metastatic castration‐resistant prostate cancer. Jpn J Clin Oncol. 2016;46:1042–1046. 27534799 10.1093/jjco/hyw110

[9] Suzuki H , Okihara K , Miyake H , et al. Alternative nonsteroidal antiandrogen therapy for advanced prostate cancer that relapsed after initial maximum androgen blockade. J Urol. 2008;180:921–927. 18635218 10.1016/j.juro.2008.05.045

[10] Okihara K , Ukimura O , Kanemitsu N , et al. Clinical efficacy of alternative antiandrogen therapy in Japanese men with relapsed prostate cancer after first‐line hormonal therapy. Int J Urol. 2007;14:128–132. 17302569 10.1111/j.1442-2042.2007.01698.x

[11] Okegawa T , Nutahara K , Higashihara E . Alternative antiandrogen therapy in patients with castration‐resistant prostate cancer: a single‐center experience. Int J Urol. 2010;17:950–955. 20807265 10.1111/j.1442-2042.2010.02620.x

[12] Nishimura K , Arichi N , Tokugawa S , Yoshioka I , Kishikawa H , Ichikawa Y . Effects of flutamide as a second‐line agent for maximum androgen blockade of hormone refractory prostate cancer. Int J Urol. 2007;14:264–267. 17430272 10.1111/j.1442-2042.2007.01681.x

[13] Miyake H , Hara I , Eto H . Clinical outcome of maximum androgen blockade using flutamide as second‐line hormonal therapy for hormone‐refractory prostate cancer. BJU Int. 2005;96:791–795. 16153202 10.1111/j.1464-410X.2005.05766.x

[14] Fujikawa K , Matsui Y , Fukuzawa S , Takeuchi H . Prostate‐specific antigen levels and clinical response to flutamide as the second hormone therapy for hormone‐refractory prostate carcinoma. Eur Urol. 2000;37:218–222. 10705202 10.1159/000020121

[15] Tran C , Ouk S , Clegg NJ , et al. Development of a second‐generation antiandrogen for treatment of advanced prostate cancer. Science. 2009;324:787–790. 19359544 10.1126/science.1168175 PMC2981508

[16] Astellas Pharma US Inc. , Medivation Inc. XTANDI US Prescribing Information, 2019. Available at: https://www.astellas.us/docs/us/12A005-ENZ-WPI.pdf. Accessed November 2020

[17] European Medicines Agency . Summary of opinion (post authorization)—Xtandi, 2018. Available at: https://www.ema.europa.eu/en/documents/smop/chmp‐post‐authorisation‐summary‐positive‐opinion‐xtandi‐ii‐39‐g_en.pdf. Accessed November 2020

[18] Astellas Pharma Inc. Xtandi® (enzalutamide) package insert—updated Japanese indication, 2020. Available at: https://pins.japic.or.jp/pdf/newPINS/00067392.pdf. Accessed November 2020

[19] Scher HI , Fizazi K , Saad F , et al. Increased survival with enzalutamide in prostate cancer after chemotherapy. N Engl J Med. 2012;367:1187–1197. 22894553 10.1056/NEJMoa1207506

[20] Akaza H , Uemura H , Tsukamoto T , et al. A multicenter phase I/II study of enzalutamide in Japanese patients with castration‐resistant prostate cancer. Int J Clin Oncol. 2016;21:773–782. 26793974 10.1007/s10147-016-0952-6 PMC4967591

[21] Beer TM , Armstrong AJ , Rathkopf DE , et al. Enzalutamide in metastatic prostate cancer before chemotherapy. N Engl J Med. 2014;371:424–433. 24881730 10.1056/NEJMoa1405095 PMC4418931

[22] Armstrong AJ , Szmulewitz RZ , Petrylak DP , et al. ARCHES: a randomized, phase III study of androgen deprivation therapy with enzalutamide or placebo in men with metastatic hormone‐sensitive prostate cancer. J Clin Oncol. 2019;37:2974–2986. 31329516 10.1200/JCO.19.00799 PMC6839905

[23] Davis ID , Martin AJ , Stockler MR , et al. Enzalutamide with standard first‐line therapy in metastatic prostate cancer. N Engl J Med. 2019;381:121–131. 31157964 10.1056/NEJMoa1903835

[24] Iguchi T , Tamada S , Kato M , Yasuda S , Yamasaki T , Nakatani T . Enzalutamide versus flutamide for castration‐resistant prostate cancer after combined androgen blockade therapy with bicalutamide: study protocol for a multicenter randomized phase II trial (the OCUU‐CRPC study). BMC Cancer. 2019;19:339. 30971225 10.1186/s12885-019-5526-3 PMC6458677

[25] Iguchi T , Tamada S , Kato M , et al. Enzalutamide versus flutamide for castration‐resistant prostate cancer after combined androgen blockade therapy with bicalutamide: the OCUU‐CRPC study. Int J Clin Oncol. 2020;25:486–494. 31564004 10.1007/s10147-019-01554-3

[26] Miyake H , Hara T , Ozono S , Fujisawa M . Impact of prior use of an androgen receptor‐axis‐targeted (ARAT) agent with or without subsequent taxane therapy on the efficacy of another ARAT agent in patients with metastatic castration‐resistant prostate cancer. Clin Genitourin Cancer. 2017;15:e217–e22. 10.1016/j.clgc.2016.07.005 27522450

[27] Eisenhauer EA , Therasse P , Bogaerts J , et al. New response evaluation criteria in solid tumours: revised RECIST guideline (version 1.1). Eur J Cancer. 2009;45:228–247. 19097774 10.1016/j.ejca.2008.10.026

[28] Scher HI , Halabi S , Tannock I , et al. Design and end points of clinical trials for patients with progressive prostate cancer and castrate levels of testosterone: recommendations of the Prostate Cancer Clinical Trials Working Group. J Clin Oncol. 2008;26:1148–1159. 18309951 10.1200/JCO.2007.12.4487 PMC4010133

[29] Beer TM , Armstrong AJ , Sternberg CN , et al. Enzalutamide in men with chemotherapy‐naive metastatic prostate cancer (mCRPC): results of phase III PREVAIL study. J Clin Oncol. 2014;32(suppl 4):LBA1–1.

[30] Narimoto K , Mizokami A , Izumi K , et al. Adrenal androgen levels as predictors of outcome in castration‐resistant prostate cancer patients treated with combined androgen blockade using flutamide as a second‐line anti‐androgen. Int J Urol. 2010;17:337–345. 20202011 10.1111/j.1442-2042.2010.02473.x

[31] Schoenfeld DA . Sample‐size formula for the proportional‐hazards regression model. Biometrics. 1983;39:499–503. 6354290

[32] Sato T . On the variance estimator for the Mantel–Haenszel risk difference. Biometrics. 1989;45:1323–1324.

