# Supplementary material for: Enzalutamide + androgen deprivation therapy (ADT) versus flutamide + ADT in Japanese men with castration‐resistant prostate cancer: AFTERCAB study
Source: BJUI Compass. 2021 Aug 20;3(1):26–36. doi: 10.1002/bco2.103 (PMC8988761; doi:10.1002/bco2.103)
Supplement: Supplementary file 1 — Data S1: Supporting information. [file BCO2-3-26-s001.pdf]

Supplementary

List of Principal Investigators for the AFTERCAB study

Koichiro Akakura, Shusuke Akamatsu, Toshiyasu Amano, Kiyohide Fujimoto, Satoshi Fukasawa, Tomoya Fukawa, Toru Harabayashi, Katsuyoshi Hashine, Taro Iguchi, Kazuyoshi Iijima, Shigeoki Ikawa, Naoki Ito, Yukio Kageyama, Masashi Kato, Kiyotaka Kawashima, Go Kimura, Hidefumi Kinoshita, Takeshi Kishida, Kazuki Kobayashi, Hiroaki Matsumoto, Hisashi Matsushima, Hideaki Miyake, Naoto Miyanaga, Yasuyoshi Miyata, Masayoshi Nagata, Motonobu Nakamura, Yuichi Nakashima, Toshinori Nishikimi, Kazuo Nishimura, Hiroyuki Onishi, Shinichi Sakamoto, Shunji Sakuma, Haruo Seki, Chikashi Seto, Yoshiyuki Shiga, Nobuaki Shimizu, Masaki Shiota, Kazuhiro Suzuki, Ken-ichi Tabata, Atsushi Takahashi, Yutaka Takezawa, Akito Terai, Hiroji Uemura, Motohide Uemura, Akira Yokomizo, Junji Yonese, Kazuhiro Yoshimura.

Supplementary Table 1 Inclusion and exclusion criteria.

**Inclusion criteria**	**Exclusion criteria**
Signed informed consent form approved by the study center institutional review board, prior to the start of the study	Severe concurrent diseases, infections, or complications, which are considered inappropriate for enrollment by the investigator
Aged ≥20 years at the time of providing informed consent	Confirmed or suspected brain metastasis or active leptomeningeal metastasis
Diagnosed with histologically or cytologically confirmed adenocarcinoma of the prostate without neuroendocrine differentiation or small-cell histology	History of malignant tumor, other than prostate cancer, in the past 5 years (except for non-melanoma skin cancer cured with radical therapy)
Receiving continuous ADT with GnRH agonist/antagonist or bilateral orchiectomy (surgical or chemical castration)	Hypersensitivity to the ingredients of enzalutamide capsules or flutamide tablets
Treatment with effective GnRH agonist/antagonist to be continued during the study period if bilateral orchiectomy is not performed	History of seizure, or any condition that may predispose to seizure
Serum testosterone level ≤1.73 nmol/L (50 ng/dL or 0.5 ng/mL) at screening	Liver disorder such as viral hepatitis and hepatic cirrhosis, or AST and ALT > ULN at screening

No change in the dose of bisphosphonate preparation or denosumab for ≥4 weeks (if used)	Receiving warfarin
Asymptomatic or mildly symptomatic prostate cancer (BFI-SF question 3 [worst pain within 24 hours] score <4)	Received treatment for prostate cancer with cytocidal chemotherapy that includes anti-androgenic agents other than bicalutamide (e.g. enzalutamide, flutamide), abiraterone, or estramustine
ECOG performance status of 0 or 1	Participated in a clinical trial on a drug other than GnRH agonist/antagonist in prostate cancer
Estimated life expectancy ≥12 months	Received treatment with herbal medications that may have hormonal anti-prostate-cancer activity or herbal medications (saw palmetto) that may decrease PSA levels
Able to swallow the study drug and comply with procedures required by the study	Received treatment for prostate cancer with systemic corticosteroids, or treatment for other diseases with systemic corticosteroids greater than the equivalent of 10 mg per day of prednisone within 4 weeks prior to enrollment
Disease progression, defined as one of the following, during CAB in the combination of bicalutamide + ADT:	Received treatment with bicalutamide within 6 weeks prior to enrollment

• PSA increase confirmed at ≥2 time points with an interval of ≥1 week; ≥6 weeks after the last dose of bicalutamide, PSA should be confirmed as higher than the highest PSA measured after the nadir was confirmed during administration of bicalutamide. PSA at screening visit should be ≥2 ng/mL (2 μg/L)<br>• Soft tissue disease progression defined by RECIST, version 1.1 [1]<br>• Progression of ≥2 bone lesions, defined as new lesions in bone scintigraphy by PCWG2 [2]	
A sexually active patient and his female partner who is of childbearing potential must use two acceptable birth control methods (one of which must include a condom as a barrier method of contraception) from screening to 3 months after the last dose of the study drug:<br>• A male or female condom as a barrier method of contraception<br>• Consistent and correct usage of established oral contraceptives	Received treatment with 5-α reductase inhibitors (finasteride, dutasteride), estrogens, or drugs with anti-tumor action other than GnRH agonists/antagonists within 4 weeks prior to enrollment

• Established intrauterine device or intrauterine system by the female partner<br>• Tubal ligation in the female partner performed ≥6 months prior to screening<br>• Vasectomy or other procedure resulting in infertility (e.g. bilateral orchiectomy) performed ≥6 months prior to screening<br>• Calendar-based contraceptive methods (Knaus-Ogino or rhythm method)	
Use of a condom throughout the study if engaging in sexual intercourse with a pregnant woman	Received treatment with opioid analgesic for pains associated with prostate cancer within 4 weeks prior to enrollment
Agreement not to donate sperm from screening to 3 months after the last dose of study drug	Participated or is currently participating in a clinical trial or a post-marketing clinical study on other ethical drugs (excluding GnRH agonist/antagonist) or medical devices within 12 weeks or 5 half-lives (in case of ethical drugs), whichever is longer, prior to screening
Agreement not to participate in another interventional study while on treatment	Received treatment for primary or metastatic lesion with surgery or radiation therapy within 4 weeks prior to enrollment

	Unstable psychiatric disease (e.g. schizophrenia, dementia, uncontrollable depression/bipolar disorder)
	Disease or reason unsuitable for participation in the study, which is considered by the investigator to cause excessive risk to the patient or make the interpretation of safety data difficult

ADT, androgen deprivation therapy; ALT, alanine aminotransferase; AST, aspartate aminotransferase; BPI-SF, Brief Pain Inventory, Short Form; CAB, combined androgen blockade; ECOG, Eastern Cooperative Oncology Group; GnRH, gonadotropin-releasing hormone; PCWG2, Prostate Cancer Working Group 2; PSA, prostate-specific antigen; RECIST, response evaluation criteria in solid tumors; ULN, upper limit of normal.

Supplementary Table 2 Full definition of efficacy endpoints.

Primary endpoint	Description
Time to PSA progression with first-line therapy (TTPP1)	Time from randomization to the date of PSA progression[a] with first-line therapy
**Secondary endpoints**	**Description**
Time to PSA progression with first-line therapy + second-line therapy (TTPP2)	Total of time to PSA progression[a] with first-line therapy (time from randomization to the date of PSA progression[a] with first-line therapy) and time to PSA progression[a] with second-line therapy (time from day 1 of second-line therapy to the date of PSA progression[a] with second-line therapy)
PSA response rate (≥50% or ≥90%) with first-line therapy	Proportion of patients who achieved PSA response[b] with first-line therapy
PSA response rate (≥50% or ≥90%) with first-line therapy at week 13	Proportion of patients who achieved PSA response at week 13[c] with first-line therapy
Time to 50% PSA reduction with first-line therapy	Time to 50% PSA reduction from baseline with first-line therapy

Time to treatment failure with first-line therapy (TTF1)	Time from randomization to discontinuation of first-line therapy for any reason.[d] Men who completed the study were considered as events at the last available assessment.
Time to treatment failure with second-line therapy (TTF2)	Time from randomization to discontinuation of second-line therapy for any reason.[d] Men who transitioned to second-line therapy and completed the study were considered as events at the last available assessment. Men who did not transition to second-line therapy were censored at the last available assessment during first-line therapy.
Radiographic progression free survival (rPFS)	Time from randomization to radiographic disease progression[e] or death from any cause during the study period, whichever occurs sooner, in men with distant metastases (M1) confirmed at baseline
**Exploratory endpoints**	**Description**
Time to PSA progression with second-line therapy (second TTPP)	Time from day 1 of second-line therapy to the date of PSA progression[a] with second-line therapy
PSA response rate (≥50% or ≥90%) with second-line therapy	Proportion of patients who achieved PSA response[b] with second-line therapy

[a]PSA progression was defined according to the PCWG2 guidelines [2]. For patients with PSA decline at week 13, PSA progression was defined as a ≥25% increase and an absolute increase of ≥2 ng/mL above the nadir, confirmed by a second consecutive value ≥3 weeks later. For patients without PSA decline at week 13, PSA progression was defined as a ≥25% increase and an absolute increase of ≥2 ng/mL above baseline; [b]PSA response was defined as a ≥50% or ≥90% reduction from baseline when ≥3 weeks passed after the lowest PSA which decreased by ≥50% or ≥90%; [c]PSA response at week 13 was defined as a ≥50% or ≥90% reduction from baseline to PSA at week 13; [d]Including disease progression, AE, patient preference, or death; [e]In patients with distant metastases at baseline, soft tissue lesions were defined according to RECIST, version 1.1 [1], and bone lesions were defined as the occurrence of ≥2 new bone lesions, confirmed by bone scintigraphy in line with PCWG2 guidelines [2]. AE, adverse event; PCWG2, Prostate Cancer Working Group 2; PSA, prostate-specific antigen; RECIST, response evaluation criteria in solid tumors.

Supplementary Fig. 1 PSA response rate (≥50% or ≥90%) with first-line therapy at week 13.

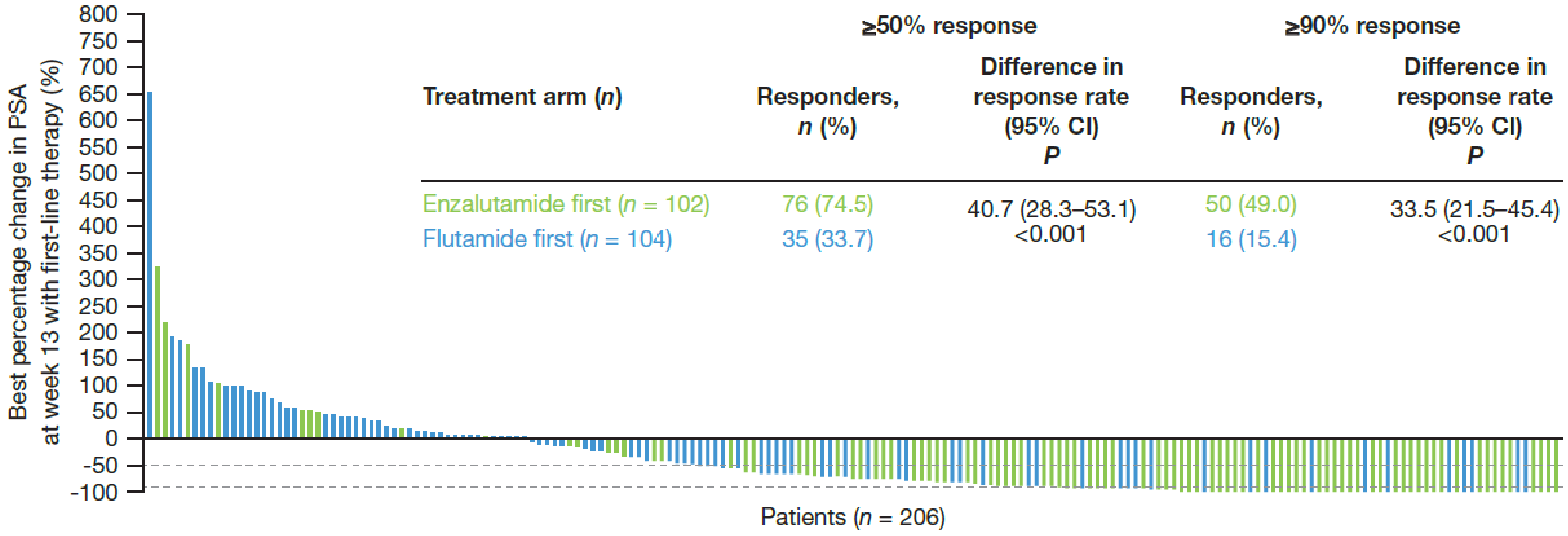

Proportion of patients who achieved PSA response at week 13 with first-line therapy. PSA response at week 13 was defined as a ≥50% or ≥90% reduction from baseline to PSA at week 13. CI, confidence interval; PSA, prostate-specific antigen.

Supplementary Fig. 2 TTF1.

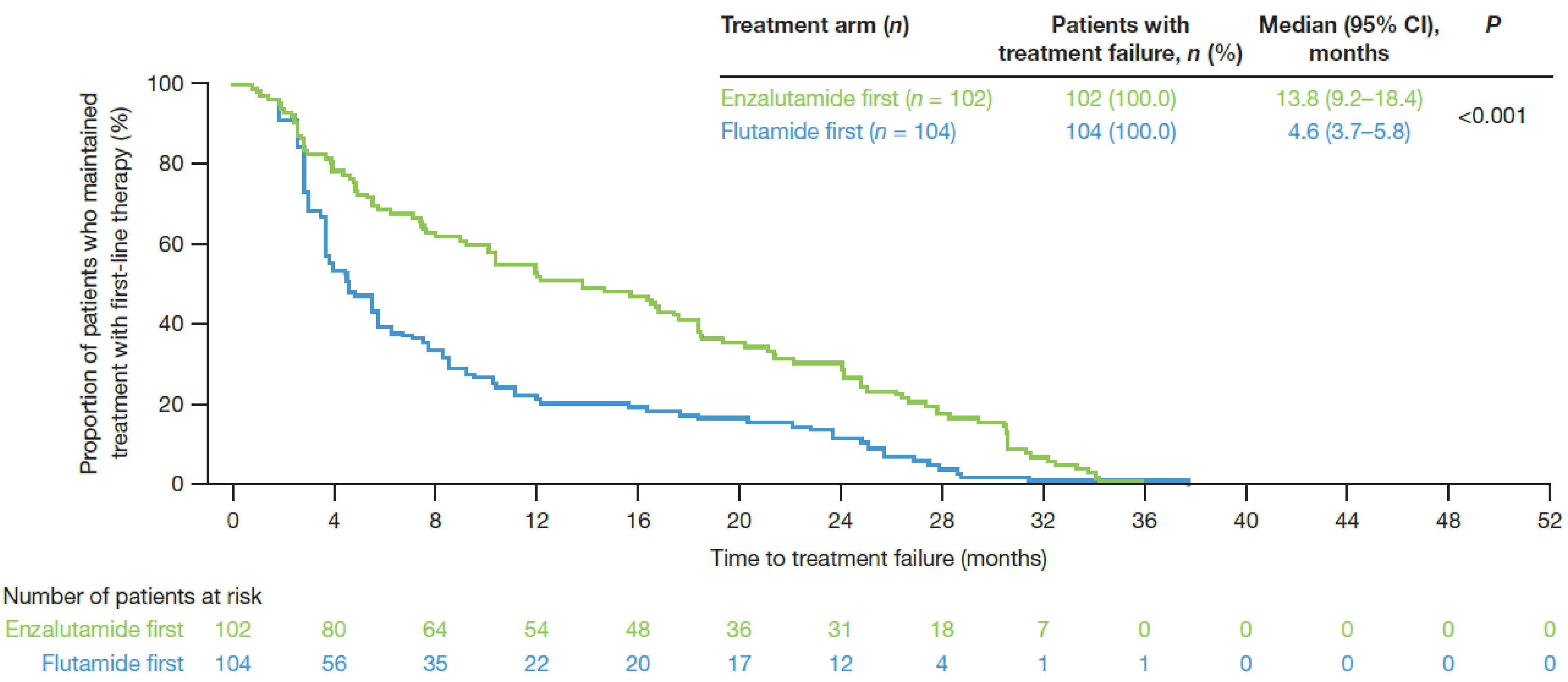

Time from randomization to discontinuation of first-line therapy for any reason, including disease progression, adverse event, patient preference, or death. Men who completed the study were considered as events at the last available assessment. CI, confidence interval; PSA, prostate-specific antigen; TTF1, time to treatment failure with first-line therapy.

Supplementary Fig. 3 TTF2.

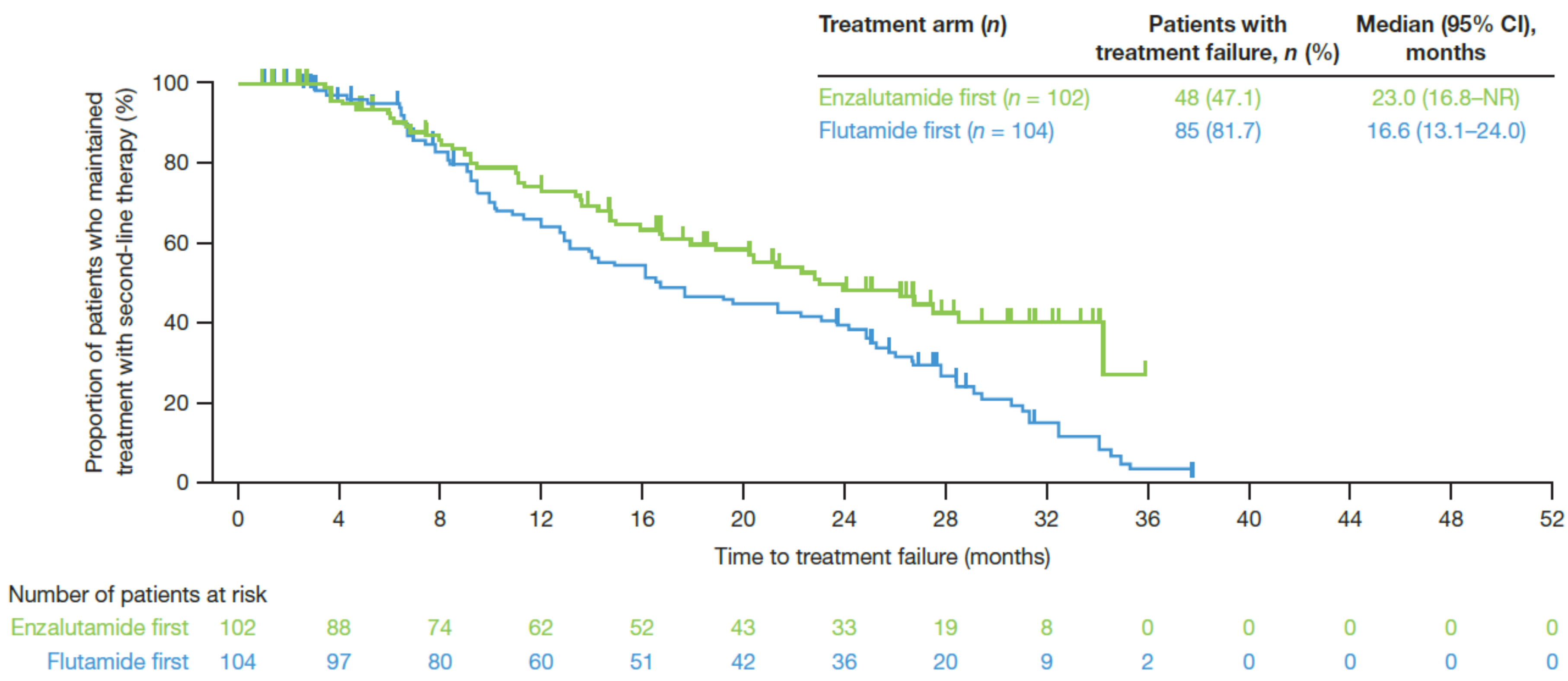

Time from randomization to discontinuation of second-line therapy for any reason, including disease progression, adverse event, patient preference, or death. Men who transitioned to second-line therapy and completed the study were considered as events at the last available

assessment. Men who did not transition to second-line therapy were censored at the last available assessment during first-line therapy. CI, confidence interval; NR, not reached; TTF2, time to treatment failure with second-line therapy.

Supplementary Fig. 4 rPFS.

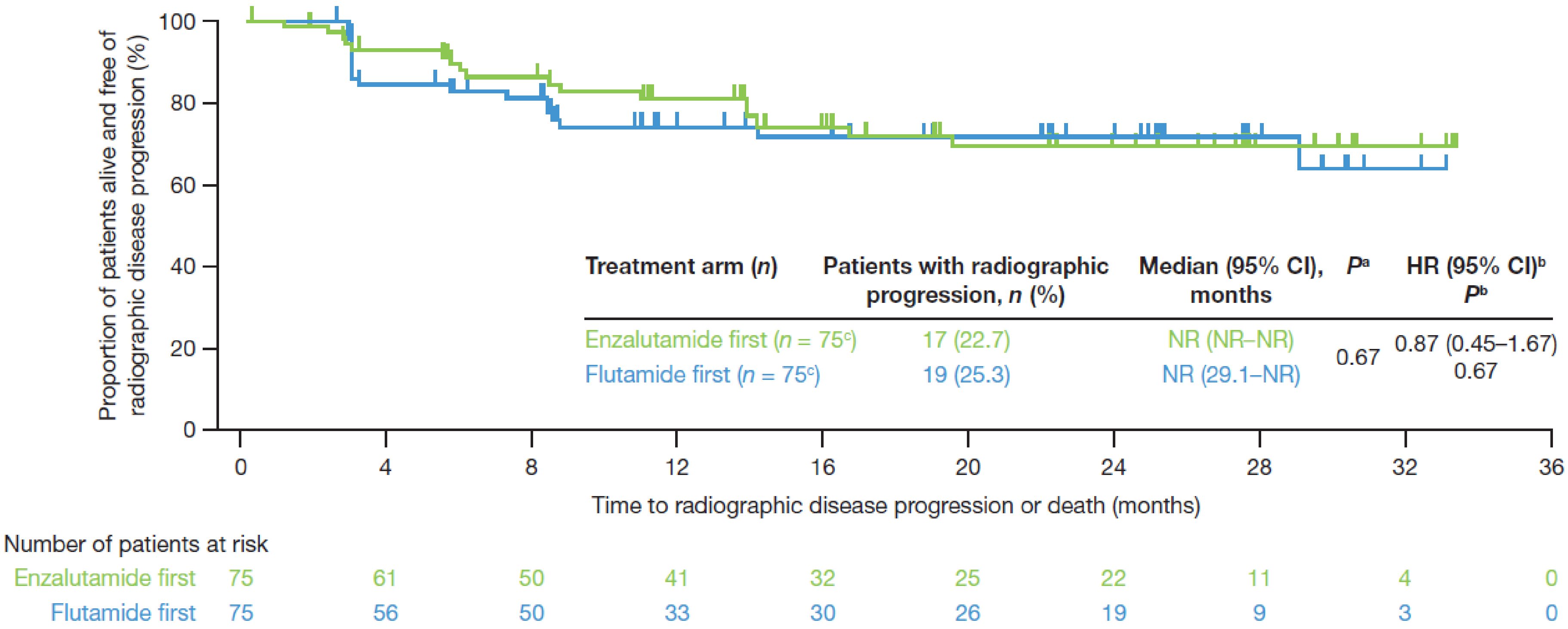

Time from randomization to radiographic disease progression or death from any cause during the study period, whichever occurs sooner, in men with distant metastases (M1) confirmed at baseline. Soft tissue lesions were defined according to RECIST, version 1.1 [1], and bone lesions were defined as the occurrence of ≥2 new bone lesions, confirmed by bone scintigraphy in line with PCWG2 guidelines [2]. [a]Stratified log-rank test,

stratified by disease stage; [b]Unstratified Cox proportional hazards model, with treatment and disease stage as covariates; [c]Men with distant metastases (M1) confirmed at baseline. CI, confidence interval; HR, hazard ratio; NR, not reached; PCWG2, Prostate Cancer Working Group 2; RECIST, response evaluation criteria in solid tumors; rPFS, radiographic progression-free survival.

Supplementary Fig. 5 Second TTPP.

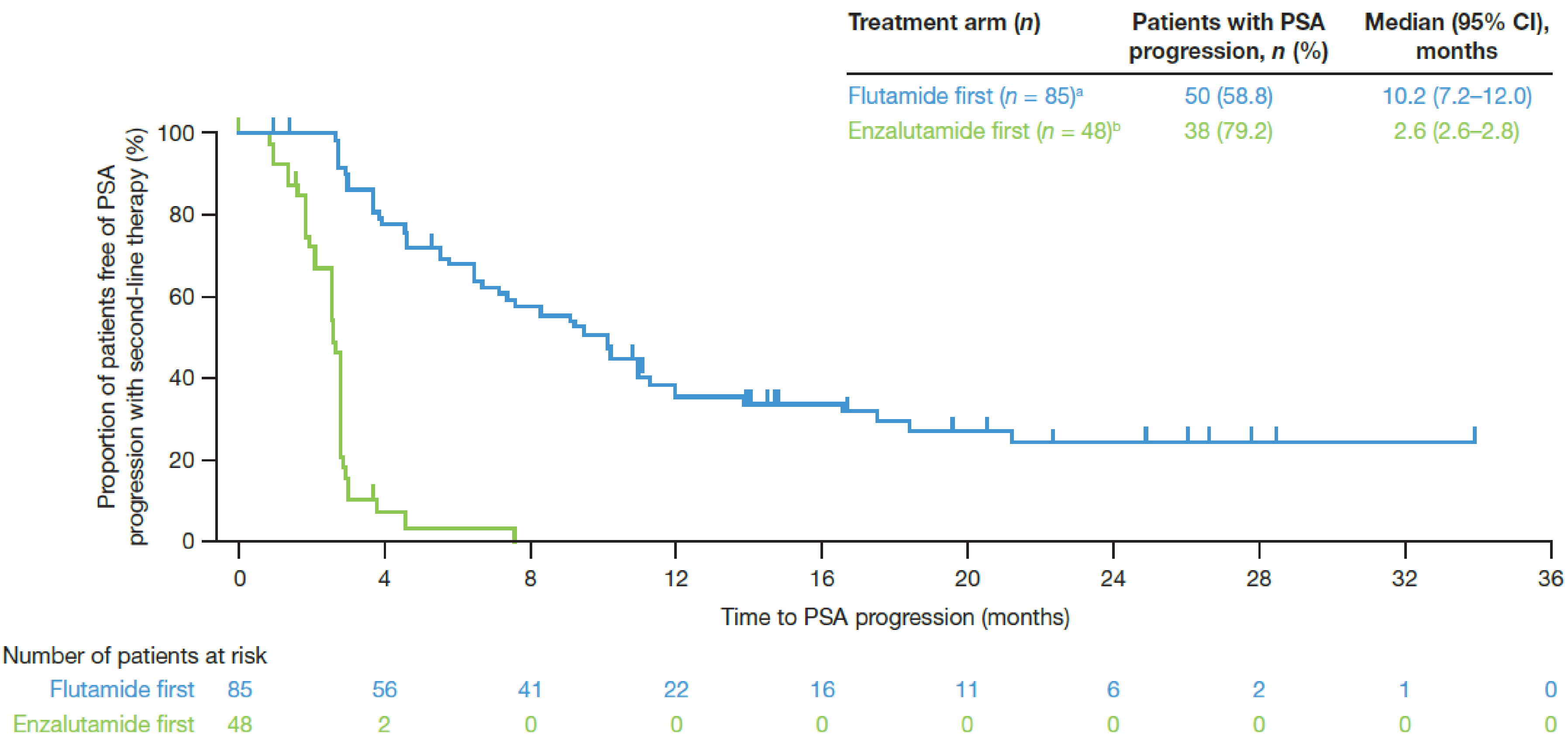

Time from day 1 of second-line therapy to the date of PSA progression with second-line therapy. PSA progression was defined according to the PCWG2 guidelines [2]. For patients with PSA decline at week 13, PSA progression was defined as a ≥25% increase and an absolute increase of

≥2 ng/mL above the nadir, confirmed by a second consecutive value ≥3 weeks later. For patients without PSA decline at week 13, PSA progression was defined as a ≥25% increase and an absolute increase of ≥2 ng/mL above baseline. [a]Men randomized to flutamide first who received enzalutamide as second-line therapy; [b]Men randomized to enzalutamide first who received flutamide as second-line therapy. CI, confidence interval; PCWG2, Prostate Cancer Working Group 2; PSA, prostate-specific antigen; RECIST, response evaluation criteria in solid tumors; TTPP, time to PSA progression.

Supplementary Fig. 6 PSA response rate (≥50% or ≥90%) with second-line therapy.

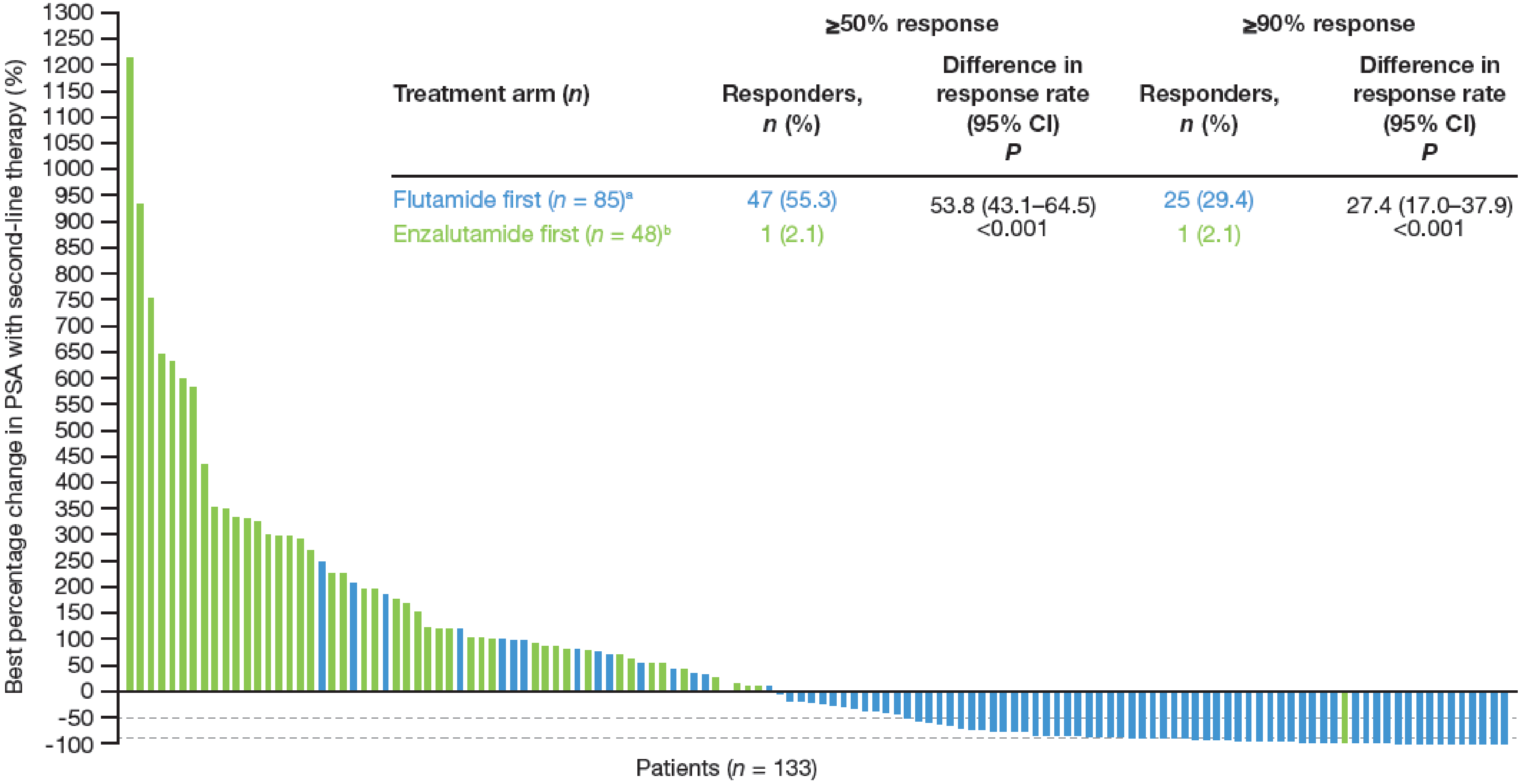

	≥50% response		≥90% response	
Treatment arm (*n*)	Responders, *n* (%)	Difference in response rate (95% CI) *P*	Responders, *n* (%)	Difference in response rate (95% CI) *P*
Flutamide first (*n* = 85)[a]	47 (55.3)	53.8 (43.1–64.5) <0.001	25 (29.4)	27.4 (17.0–37.9) <0.001
Enzalutamide first (*n* = 48)[b]	1 (2.1)		1 (2.1)	

Proportion of patients who achieved PSA response with second-line therapy. PSA response was defined as a ≥50% or ≥90% reduction from baseline when ≥3 weeks passed after the lowest PSA which decreased by ≥50% or ≥90%. [a]Men randomized to flutamide first who received enzalutamide as second-line therapy; [b]Men randomized to enzalutamide first who received flutamide as second-line therapy. CI, confidence interval; PSA, prostate-specific antigen.

References

[1] **Eisenhauer EA, Therasse P, Bogaerts J et al.** New response evaluation criteria in solid tumours: revised RECIST guideline (version 1.1). *Eur J Cancer* 2009; 45: 228–47

[2] **Scher HI, Halabi S, Tannock I et al.** Design and end points of clinical trials for patients with progressive prostate cancer and castrate levels of testosterone: recommendations of the Prostate Cancer Clinical Trials Working Group. *J Clin Oncol* 2008; 26: 1148–59
